# Involvement of a Nonstructural Protein in Poliovirus Capsid Assembly

**DOI:** 10.1128/JVI.01447-18

**Published:** 2019-02-19

**Authors:** Oluwapelumi O. Adeyemi, Lee Sherry, Joseph C. Ward, Danielle M. Pierce, Morgan R. Herod, David J. Rowlands, Nicola J. Stonehouse

**Affiliations:** a School of Molecular and Cellular Biology, Faculty of Biological Sciences, University of Leeds, Leeds, United Kingdom; University of Texas Southwestern Medical Center

**Keywords:** 2A^pro^, cleavage, evolution, poliovirus, selection

## Abstract

Virus capsid proteins must perform a number of roles. These include self-assembly and maintaining stability under challenging environmental conditions, while retaining the conformational flexibility necessary to uncoat and deliver the viral genome into a host cell. Fulfilling these roles could place conflicting constraints on the innate abilities encoded within the protein sequences. In a previous study, we identified a number of mutations within the capsid-coding sequence of poliovirus (PV) that were established in the population during selection for greater thermostability by sequential treatment at progressively higher temperatures. Two mutations in the VP1 protein acquired at an early stage were maintained throughout this selection procedure. One of these mutations prevented virion assembly when introduced into a wild-type (wt) infectious clone. Here we show, by sequencing beyond the capsid-coding region of the heat-selected virions, that two mutations had arisen within the coding region of the 2A protease. Both mutations were maintained throughout the selection process. Introduction of these mutations into a wt infectious clone by site-directed mutagenesis considerably reduced replication. However, they permitted a low level of assembly of infectious virions containing the otherwise lethal mutation in VP1. The 2A^pro^ mutations were further shown to slow the kinetics of viral polyprotein processing, and we suggest that this delay improves the correct folding of the mutant capsid precursor protein to permit virion assembly.

**IMPORTANCE** RNA viruses, including poliovirus, evolve rapidly due to the error-prone nature of the polymerase enzymes involved in genome replication. Fixation of advantageous mutations may require the acquisition of complementary mutations which can act in concert to achieve a favorable phenotype. This study highlights a compensatory role of a nonstructural regulatory protein, 2A^pro^, for an otherwise lethal mutation of the structural VP1 protein to facilitate increased thermal resistance. Studying how viruses respond to selection pressures is important for understanding mechanisms which underpin emergence of resistance and could be applied to the future development of antiviral agents and vaccines.

## Abstract (precis)

RNA viruses, including poliovirus, evolve rapidly due to the error-prone nature of the polymerase enzymes involved in genome replication. Fixation of advantageous mutations may require the acquisition of complementary mutations which can act in concert to achieve a favorable phenotype. This study highlights a compensatory role of a nonstructural regulatory protein, 2A^pro^, for an otherwise lethal mutation of the structural VP1 protein to facilitate increased thermal resistance. Studying how viruses respond to selection pressures is important for understanding mechanisms which underpin emergence of resistance and could be applied to the future development of antiviral agents and vaccines.

## INTRODUCTION

RNA viruses have high mutation rates that contribute to population diversity, genetic robustness, and ability to withstand population bottlenecks (1, 2). The RNA-dependent RNA polymerase enzyme (RdRp) lacks a proofreading function, thereby resulting in error-prone replication. As a result, RNA viruses exist as swarms of variants, also known as quasispecies. As viruses adapt under altered growth constraints, mutants with a replicative advantage in the face of selection emerge from the quasispecies and alter the population sequence composition. However, this may occur at a cost to virus fitness (1, 3).

**FIG 1:**
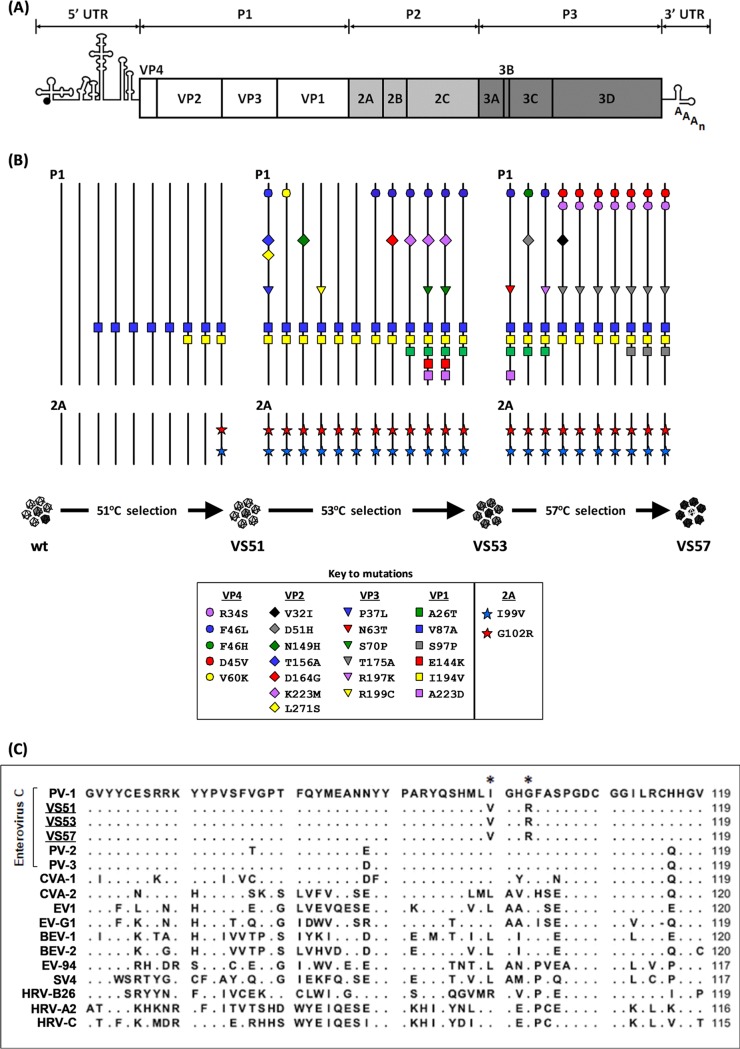
Identification of thermally selected mutations. (A) Cartoon structure of the PV genome, showing the 5′ and 3′ UTRs flanking the open reading frame comprising the structural (P1) and nonstructural (P2 and P3) regions. (B) Evolution of PV-1 under thermal selection. Viral RNA was extracted from each passage of virions selected at 51°C (i.e., VS51), 53°C (i.e., VS53), and 57°C (i.e., VS57) (25). The entire genome of the evolving population at each passage was reverse transcribed and amplified by PCR. The virus pool was sequenced and aligned against the wt PV-1 sequence by ClustalOmega. A cartoon representation of selected mutations is shown. Solid black vertical lines represent wt sequences of the P1 and 2A regions. Nonsynonymous mutations are presented as colored shapes in VP4, VP2, VP3, VP1, and 2A^pro^, as shown in the key inset. (C) Sequence comparisons of 2A^pro^ among enteroviruses. Alignment of a 60-residue region of 2A^pro^ of thermally selected viruses VS51, VS53, and VS57 against enterovirus A (coxsackievirus A1 [CVA-1]), enterovirus B (echovirus 1 [EV1]), enterovirus C (PV-1, PV-2, PV-3, and CVA-2), enterovirus D: human enterovirus 94 (EV-94); enterovirus E: bovine enterovirus (BEV-1); enterovirus F (BEV-2), enterovirus G (porcine enterovirus G1 [EV-G1]), enterovirus H (simian enterovirus SV4 [SV4]), rhinovirus A (human rhinovirus A [HRV-A]), rhinovirus B (HRV-B), and rhinovirus C (HRV-C). Thermally selected virions VS51, VS53, and VS57 are underlined. Enterovirus C members are annotated with a bracket. The 2A^pro^ consensus residues of wt PV-1 are shown in bold. Matching residues are shown as dots beneath the corresponding residues of wt PV-1. Variable residues are shown underneath the corresponding positions of the wt PV-1 residues. Asterisks on the consensus sequence indicate positions that correspond to residues I99 and G102, respectively. Sequences were aligned using the default alignment algorithms of CLC sequencing viewer version 6.

Poliovirus (PV), which occurs as three serotypes, PV-1 to -3, is a picornavirus with a 7.5-kb positive-sense, single-stranded RNA genome enclosed within a 30-nm icosahedral capsid. The genome (Fig. 1A) comprises a coding region that is flanked at the 5′ and 3′ ends by untranslated regions (UTR) (4). Upon cell entry, the coding region is translated into a polyprotein precursor which is cleaved by viral proteases, 2A^pro^ and 3C^pro^ (or its precursor 3CD^pro^). Upon translation, the structural precursor protein, P1, is autocatalytically cleaved from the polyprotein by 2A^pro^ (5), and P1 is further processed into VP1, VP3, and VP0 by 3C^pro^/3CD^pro^. The two nonstructural precursors, P2 and P3, are processed into 2A^pro^, 2B, and 2C and into 3A, 3B, 3C^pro^, and 3D^pol^, respectively, again by 3C^pro^/3CD^pro^ (6, 7). Replication of the PV genome is mediated by the RdRp 3D^pol^ (8) and primed by 3B within a membrane-bound complex that also includes 2B and 2C (9, 10) and 3A (11), whose roles are reviewed in reference 12.

The PV 2A^pro^ is a cysteine protease that has been described as a multifunctional regulatory protein due to its roles at various stages of the viral life cycle (reviewed in reference 13). It has an active site that comprises a catalytic triad (H20, D38, and C109) (14, 15), as well as highly conserved cysteine and histidine residues (C55, C57, C115, and H117) that have been shown to be critical for maintaining structural integrity and for *cis*- and *trans*-catalytic activities (16, 17). The PV 2A^pro^ has been shown to stimulate internal ribosome entry site (IRES)-mediated viral translation over cap-dependent translation of host mRNA through inactivation of a key host cellular translation initiation factor, eukaryotic initiation factor 4G (eIF4G) (18–20). It has also been shown to have a role in the control of genome replication by stimulating negative-strand RNA synthesis and enhancing RNA stability (21, 22). 2A^pro^ also plays a key role in virion assembly (5) by cleaving the P1 region from the polyprotein. This is followed by the self-assembly of VP0, VP3, and VP1 into genome-free capsids or around the nascent genome to form virion particles in which VP0 is cleaved into VP2 and VP4. It has been shown for the attenuated Sabin vaccine strains of PV-2 and PV-3 that 2A^pro^ can compensate for cell-specific attenuating mutations within the 5′ UTR (23). Additionally, a study recently reported on compensatory 2A^pro^ mutations within acid-resistant enterovirus D94 variants that evolved capsid-stabilizing mutations (24). Together, these findings suggest important roles for 2A^pro^ in the evolution of picornaviruses; however, the mechanisms responsible are unclear.

We recently described the evolution of heat-resistant PV-1 capsids through multiple cycles of thermal selection (25). Here, we characterized mutations within the nonstructural protein of the thermally selected virus populations and described the mechanism by which these facilitated the correct assembly of the heat-resistant capsids.

## RESULTS

### Analysis of a population of PV-1 evolving during sequential thermal stressing.

Previously, we employed *in vitro* selection of PV-1 by sequential heating in order to select mutations in the viral structural proteins that increased the thermal stability of the capsid (25). Here, we determined the consensus sequence of the evolving population at each passage during the selection (Fig. 1B). VP1-V87A was the first capsid mutation observed within the population, following three selection cycles at 51°C. After five further cycles of selection at 51°C, VP1-I194V also appeared, after which both mutations were maintained in the consensus sequences of all subsequent virus populations selected at 51°C (VS51), 53°C (VS53), and 57°C (VS57). We introduced each of the capsid protein mutations identified in VS51, VS53, and VS57 (Fig. 1B) into an infectious clone of PV-1 and showed that VP1-I194V alone prevented virus assembly. For these experiments *in vitro*-generated T7 RNA transcripts of the mutated genomes were transfected into mouse L cells, and the harvested virions were titrated using HeLa cells (Table 1). The use of L cells (which do not possess the PV receptor) ensured single-cycle infection.

**TABLE 1:** Recovery of infectious virions with mutations in structural proteins

Capsid mutation^*a*^	Mutant construct^*b*^	Recovery of infectious virion
VP1-A26T	pT7Rbz-PV1-VP1_A26T_	Yes
VP1-V87A	pT7Rbz-PV1-VP1_V87A_	Yes
VP1-S97P	pT7Rbz-PV1-VP1_S97P_	Yes
VP1-I194V	pT7Rbz-PV1-VP1_I194V_	No
VP3-C175A	pT7Rbz-PV1-VP3_C175A_	Yes
VP4-R34S	pT7Rbz-PV1-VP4_R34S_	Yes
VP4-D45V	pT7Rbz-PV1-VP4_D45V_	Yes
VP4-F46L	pT7Rbz-PV1-VP4_F46L_	Yes

a Structural mutations identified in previously reported thermally selected viruses (34).

b Capsid mutations were individually introduced into cDNA (pT7Rbz-PV1) by site-directed mutagenesis. T7 RNA transcripts were transfected into mouse L cells, and infectious virions recovered were titrated by plaque assays using HeLa cells (*n* = 3).

We extended our sequence analyses beyond P1 and showed that in addition to the capsid mutations identified in VS51, two nonstructural mutations were identified within the 2A^pro^ region of the genome (i.e., 2A-I99V and 2A-G102R) and were maintained in further rounds of thermal selection (Fig. 1B). No other nonsynonymous mutations were identified within the populations; however, synonymous mutations were identified in VS53 (i.e., 2A-G95 [GGC/GGA] and 2A-G101 [GGC/GGG]) and VS57 (i.e., 2C- V47 [GTA/GTG]). The level of conservation of the two substituted 2A^pro^ residues among enterovirus species was investigated through the alignment of 2A^pro^ reference sequences of representative members, i.e., enteroviruses A, B, C, D, E, F, G, and H and rhinoviruses A, B, and C. This showed that 2A-I99 is highly conserved among enterovirus C species but varies among other enteroviruses, while 2A-G102 is highly conserved across all enterovirus species (Fig. 1C). Because 2A^pro^ is a multifunctional protein that is known to play important roles during translation (18–20), replication (21, 22), and morphogenesis (5, 26) of PV, we investigated the significance of the mutations selected in 2A^pro^ during adaptation to thermal stress.

### Effects of 2A-I99V and 2A-G102R on the *cis* cleavage activity of 2A^pro^.

**FIG 2:**
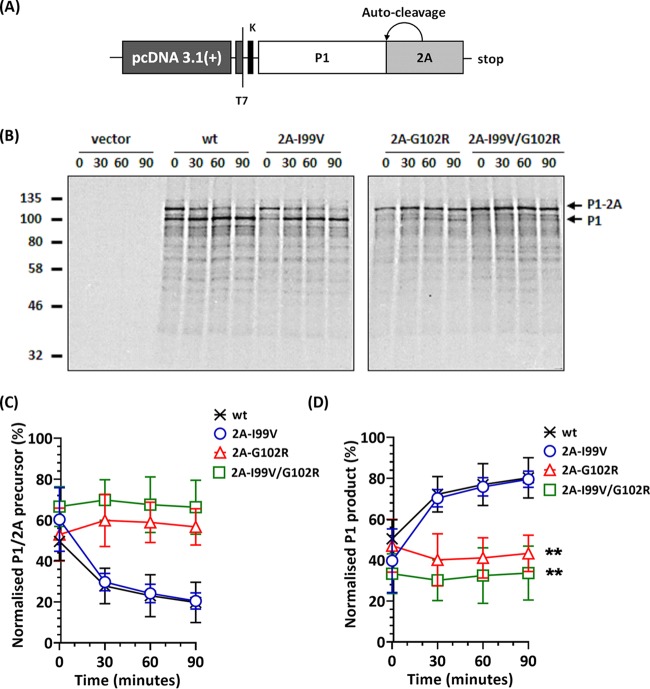
Effect of 2A^pro^ mutations on *cis*-cleavage activity at the P1/2A junction. (A) Cartoon of the construct used in the transcription/translation (T_N_T) assay. (B) Autoradiographs of SDS-PAGE of T_N_T samples. Following incubation at 30°C for 90 min, further incorporation of [^35^S]Cys-Met was prevented by the addition of excess unlabeled Cys-Met and samples collected at 30-minute intervals. Samples were separated by SDS-PAGE and protein bands detected by autoradiography. Arrows correspond to the P1/2A^pro^ precursor and processed P1. Time points represent chase. (C and D) Normalized densitometry of the P1/2A^pro^ precursor (C) and cleaved P1 (D) over time. Graphs represent the intensity of the P1 band of phosphoscreen scans of autoradiograph. Scanned images were analyzed by ImageJ version 1.47t (*n* = 2; error bars show standard errors of the means [SEM]; **, *P* < 0.001 compared to wt).

The structure of 2A^pro^ (27) comprises a catalytic triad (14, 15) and highly conserved cysteine and histidine residues that maintain the catalytic activity and structural integrity of 2A^pro^, respectively (16, 28). Neither of the 2A^pro^ mutations identified here involved these residues; however, proximity to a catalytic residue (C109) could affect activity (13–16, 26). 2A^pro^ has been shown to autocatalytically cleave the viral polyprotein in *cis* between tyrosine and glycine residues at the P1/2A junction (26, 28–31). To investigate this cleavage by the mutant versions of 2A^pro^, nonreplicative subgenomic constructs of the P1 region with 2A (i.e., P1-2A) were cloned into a pcDNA 3.1(+) vector (Fig. 2A) and termed pcDNA-P1/2A, pcDNA-P1/2A_I99V_, pcDNA-P1-2A_G102R_, and pcDNA-P1/2A_I99V/G102R_. These constructs were expressed using an *in vitro* coupled transcription/translation (T_N_T) system under the transcriptional control of a T7 promoter and translated in the presence of [^35^S]Cys-Met for 90 min at 30°C. Excess unlabeled Cys-Met was then added to prevent further ^35^S incorporation. Samples were harvested at 30-minute intervals to assess *cis* cleavage of the P1-2A subgenomic precursor.

We confirmed that the wild-type (wt) precursor P1/2A and also the 2A-I99V construct efficiently self-processed to produce P1 and 2A. However, processing of the 2A-G102R construct was severely restricted when this mutation was present individually or in combination with 2A-I99V (Fig. 2B). Quantitative analyses of the autoradiographs were undertaken, in comparison with processing of the wt proteins. The data showed that 2A-G102R either alone or in combination with 2A-I99V reduced the amount of processed P1/2A precursor (Fig. 2C), by 46% and 58%, respectively. The amount of P1 product from 2A-G102R alone or combination with 2A-I99V was similarly reduced by 46% and 58%, respectively, over the wt levels (Fig. 2D).

### Effects of 2A^pro^ mutations (I99V/G102R) on PV-1 polyprotein processing.

**FIG 3:**
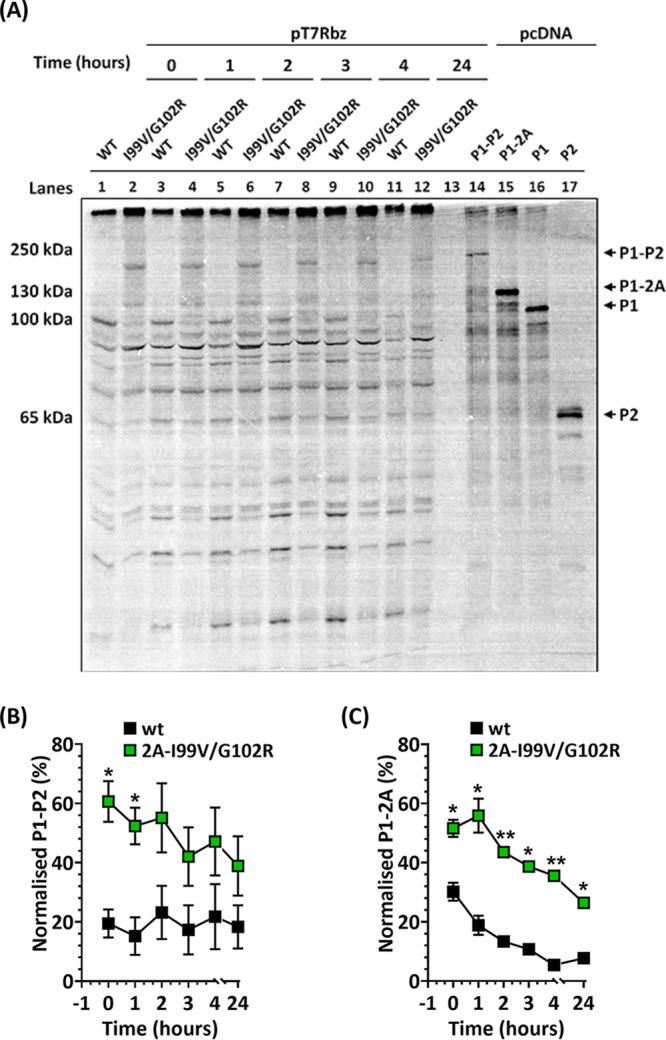
Polyprotein processing of wt and 2A^pro^ mutant PV-1. (A) Both 2A mutations were introduced into an infectious clone of wt PV-1 (i.e., pt7Rbz). RNA transcripts of pt7Rbz or 2A-I99V/-G102R were used in HeLa cell-free reactions. Following incubation at 34°C for 2 h, excess Cys-Met was added and samples taken at various time points. Samples (lanes 1 to 12) were separated by 8% SDS-PAGE and radiolabeled proteins detected by autoradiography. To identify specific bands, pcDNA constructs of P1, P2, noncleavable P1-2A, and noncleavable P1-P2 were expressed in T_N_T reactions in the presence of [^35^S]Cys-Met and incubated for 3 h (lanes 13 to 17). (B and C) Band intensities were quantified from a phosphoscreen image and the levels of P1-P2 (B) and P1-2A (C) presented as normalized percent intensity over background phosphorescence. Scanned images were analyzed by ImageJ version 1.47t (*n* = 3; error bars show SEM; *, *P* < 0.05; **, *P* < 0.001 **[**compared to wt at each time point]).

Since the proteolytic activity of 2A^pro^ on the truncated P1-2A polyprotein was affected by the selected 2A^pro^ mutations (Fig. 2), we investigated downstream effects of the 2A^pro^ mutations on processing of the full-length viral polyprotein using a HeLa cell-free system (32, 33). A combination of both 2A^pro^ mutations was introduced into cDNA clone pT7RbzPV1 using site-directed mutagenesis (SDM) to create pT7RbzPV1-2A_I99V/G102R_. RNA transcripts of pT7RbzPV1 or pT7RbzPV1-2A_I99V/G102R_ were translated for 2 h in a HeLa cell-free lysate in the presence of [^35^S]Cys-Met. Following a chase with excess unlabeled Cys-Met, polyprotein processing was assayed by SDS-PAGE and autoradiography as shown in lanes 1 to 12 of Fig. 3A. To help identify cleavage products, we generated predefined proteins from pcDNA 3.1(+) vectors. Noncleavable P1-P2 (termed pcDNA-P1-P2) and P1-2A (termed pcDNA-P1-2A) were generated by incorporating the mutation 2A-C109A, which ablates catalytic activity of 2A^pro^ (14). Additionally, subgenomic constructs of P1 only (termed pcDNA-P1) and P2 only (termed pcDNA-P2) were generated. Subgenomic constructs inserted into pcDNA 3.1(+) vectors were expressed in T_N_T assays and incubated at 30°C for 3 h in the presence of [^35^S]Cys-Met. The expressed proteins were used as markers to identify protein band patterns produced by polyprotein processing as shown in lanes 14 to 17 of Fig. 3A.

As shown in lanes 1, 3, 5, 7, 9, and 11 of Fig. 3A, the processing profile and kinetics for the wt polyprotein followed an expected pattern, with cotranslational release of P1 through autocatalytic cleavage by 2A^pro^, followed by the processing of P2-P3 and further processing of P2 and P3 (6, 26). In contrast, the reduced autocatalytic processing of the polyprotein produced from the mutant construct pT7RbzPV1-2A_I99V/G102R_ resulted in a delayed appearance of several intermediate products, and two large subgenomic precursor proteins (Fig. 3A, lanes 2, 4, 6, 8, 10 and 12) with sizes similar to those of uncleaved P1-P2 (lane 14) and P1-2A (lane 15) were detected. To determine processing rates, band intensities were analyzed using ImageJ. A precursor corresponding to P1-P2, which was seen in mutant 2A-I99V/G102R but not in the wt, was slowly processed over 24 h (Fig. 3B). A precursor corresponding to P1-2A, which could be detected in the wt but was more evident in mutant 2A-I99V/G102R, was processed more slowly in the latter (Fig. 3C). This suggested that 3C_pro_/3CD_pro_ processing of these precursors was less efficient in the mutant 2A-I99V/G102R.

### Effects of 2A^pro^ mutations (I99V/G102R) on genome replication.

**FIG 4:**
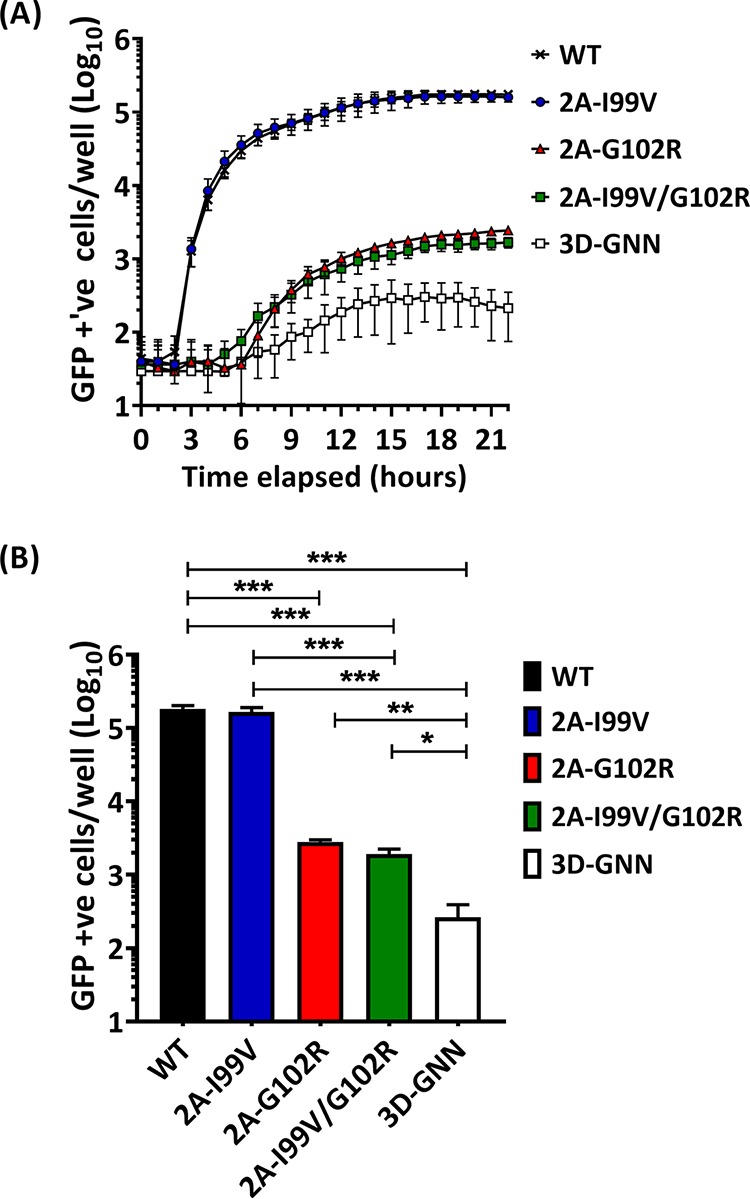
Effect of 2A^pro^ mutations on PV-1 replicon replication. (A) HeLa cells were transfected with T7 RNA transcripts and replication monitored by GFP fluorescence over time using an IncuCyte Zoom. A replication-deficient mutant, 3D-GNN, was included as a control for input translation. (B) The data from panel A at 22 h posttransfection (total GFP**-**positive cells) were also plotted as a bar graph for clarity (*n* = 3; error bars show SEM; *, *P* < 0.05; **, *P* < 0.001; ***, *P* < 0.0001).

It has been reported that although 2A^pro^ has no direct effect on positive-strand RNA synthesis, it has a stimulatory role in negative-strand synthesis and thereby could regulate RNA replication (21). We therefore investigated the effects of the 2A^pro^ mutations on viral replication using a modified version of cDNA clone pT7RbzPV-1. Here, the P1 capsid precursor was replaced with the green fluorescent protein (GFP)-coding sequence from Ptilosarcus gurneyi, creating a subgenomic replicating replicon, termed pRepPV1 (Fig. 4A). Both 2A^pro^ mutations were introduced into pRepPV1 individually (to create pRepPV1-2A_I99V_ and pRepPV1-2A_G102R_) or in combination (to create pRepPV1-2A_I99V/G102R_). A replication-deficient construct with a double point mutation (GDD to GNN) in the 3D^pol^ active site (34) was used as a control for input translation. *In vitro*-transcribed RNAs were generated from the replicon constructs and transfected into HeLa cells. Replication kinetics were monitored in real time using an IncuCyte Zoom system as described in Materials and Methods. The data are shown in Fig. 4A, with endpoint data shown in Fig. 4B for clarity. The 2A-I99V replicon replicated at levels similar to those of the wt; however, replication of the 2A-G102R or 2A-I99V/G102R replicon was approximately 100-fold lower than that of the wt (*P* < 0.0001), although it was still 10-fold higher than the input translation levels of the replication-deficient GNN replicon (*P* < 0.05). The rate of replication of 2A-I99V was similar to that of the wt, but there was a lag for 2A-G102R and 2A-I99V/G102R. This shows that the presence of the 2A-G102R mutation resulted in a significant reduction in RNA replication.

### The 2A^pro^ mutations can rescue assembly-defective capsid mutations.

In our previous report, we showed that populations of PV-1 thermally selected at 51°C possessed two common VP1 mutations (i.e., I194V and V87A) (25), both of which were maintained through further selection cycles. We introduced both mutations individually into pT7Rbz-PV1 and showed that VP1-V87A was compatible with the production of infectious virions; however, the construct with VP1-I194V could not assemble infectious particles (25). Since both 2A^pro^ mutations were propagated alongside the VP1 mutations during selection (Fig. 1B), we investigated here whether the 2A^pro^ mutations could rescue the assembly-deficient phenotype of the VP1-I194V mutant.

Infectious clones of PV-1 (pT7Rbz-PV1) with the 2A^pro^ mutations I99V/G102R (pT7Rbz-PV1-2A_I99V/G102R_) or the assembly-deficient mutation VP1-I194V alone (pT7Rbz-PV1-VP1_I194V_) or in combination with the 2A^pro^ mutations (pT7Rbz-PV1_I194V_-2A_I99V/G102R_) were generated. T7 RNA transcripts of all four constructs were transfected into HeLa cells or mouse L cells. After 24 h of incubation at 37°C, virus particles were harvested by freeze-thawing of cells and clarification of the supernatants. Titers of infectious virions harvested from both transfected cell lines were determined by plaque assays using HeLa cells, while thermal stabilities of the recovered virions were assessed as previously described (25).

**FIG 5:**
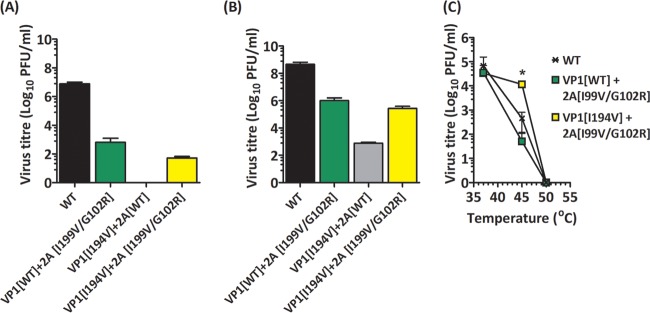
Effects of 2A^pro^ mutations on virus recovery. RNA transcripts were generated from infectious clones of the wt or VP1-I194V in the presence or absence of the 2A^pro^ mutations 2A-I99V/-G102R, transfected into mouse L cells or HeLa cells, and incubated at 37°C for 24 h. (A) Virus titers recovered from transfected mouse L cells (*n* = 3; error bars show SEM; ***, *P* < 0.0001) (B) Virus titers recovered from transfected HeLa cells. (*n* = 3; error bars show SEM; ***, *P* < 0.0001) (C) Virus samples recovered from HeLa cells were diluted in serum-free medium to equal starting titers, incubated at a range of temperatures between 37°C and 55°C for 30 min, cooled to 4°C, and titrated by plaque assays using HeLa cells (*n* = 2; error bars show standard deviations [SD]; *, *P* < 0.05 compared to wt).

The PV-1 titers from L cells and HeLa cells transfected with the construct containing the 2A-I99V/G102R mutations were reduced by 5 log_10_ PFU/ml (Fig. 5A) and 3 log_10_ PFU/ml (Fig. 5B) in comparison with wt, respectively. In the L cells, VP1-I194V alone did not produce infectious virions, as expected. It should be noted that L cells lack the PV receptor and therefore support only single-cycle infection. Therefore, virions produced in HeLa cells were possible revertants amplified through cell-to-cell spread. Our data further showed that VP1-I194V in combination with 2A-I99V/G102R resulted in infectious virion titers in both L cells and HeLa cells that were similar to those of infectious clones with wt P1 and both 2A^pro^ mutations. Furthermore, the thermal inactivation profile of the recovered virus showed that VP1-I194V-2A-I99V/G102R was more thermally stable than the wt or 2A-I99V/G102R (Fig. 5C). Together, these data suggest that the mutation VP1-I194V provided thermal stability to the viral capsid, as expected (25), and that the 2A mutations allowed rescue of this mutant.

To assess the genetic stability of the population, virus mutant VP1-I194V-2A-I99V/G102R was passaged in the absence of selection pressure using HeLa cells until viral titers were equivalent to those of the wt. Sequencing of these “restored” virions showed that the 2A^pro^ second-site (compensatory) mutations had reverted in the absence of selection pressure (data not shown).

### 2A^pro^ mutations do not act by reducing translation.

Assembly of icosahedral viral capsids is a complex process which is not yet fully understood. A study with the plant virus brome mosaic virus suggested that slower translation of the capsid proteins could result in enhanced assembly (35). In view of these observations, we investigated the consequences of reducing the translation efficiency of the PV-1 infectious clone incorporating the VP1-I194V mutation on the recovery of virions.

**FIG 6:**
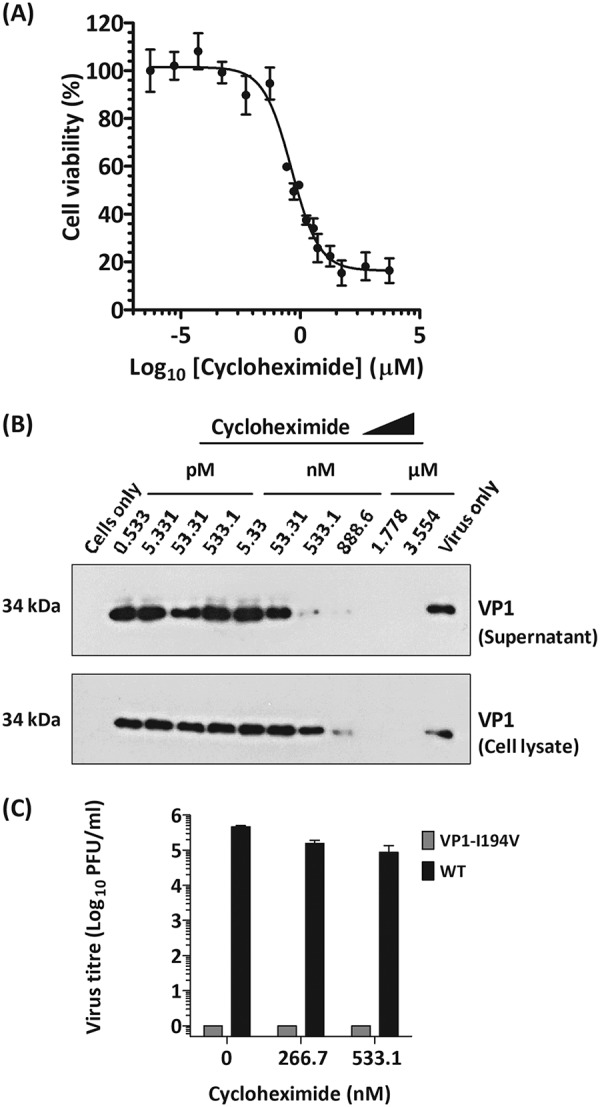
Effects of reduced translation on an assembly-deficient VP1-I194V mutant. (A) Mouse L cells were treated with increasing concentrations of cycloheximide and assayed for toxicity by using MTS. The IC_50_ was evaluated by using dose-dependent curves (*n* = 2; error bars show SD). (B) Mouse L cells were transfected with T7 RNA transcripts of the wt and treated with cycloheximide at increasing concentrations. Cell lysates were harvested using RIPA buffer. Supernatants and cell lysates were separated by SDS-PAGE and immunoblotted against anti-PV-1 VP1 MAb 8560. Representative results from two biological repeats are shown. (C) Mouse L cells were transfected with T7 RNA transcripts of the wt and VP1-I194V mutant and treated at two concentrations of cycloheximide. Supernatants were clarified by low-speed centrifugation. Virus titers of supernatants from cycloheximide-treated wt- and VP1-I194V mutant-transfected cells were determined by plaque assays using HeLa cells (*n* = 2; error bars show SEM; *, *P* < 0.05; ***, *P* < 0.0001 [compared to untreated wt]).

Cycloheximide is known to inhibit eukaryotic protein synthesis by stopping ribosomal elongation during translation (36), and micromolar concentrations of cycloheximide have been shown to shut off PV-1 translation in HeLa cells (37, 38). To determine whether partial inhibition of translation could replicate the assembly compensatory effects of the 2A^pro^ mutations, we investigated the effect of reducing/slowing translation of PV-1 in L cells using low concentrations of cycloheximide. First, we used conventional cytotoxicity assays to demonstrate a 50% inhibitory concentration (CC_50_) of 420 nM in mouse L cells (Fig. 6A). To determine concentrations at which PV translation can be reduced but not eliminated, transfected mouse L cells were treated with increasing concentrations of cycloheximide at 1.5 h posttransfection and incubated at 37°C for 24 h. Cell lysates and supernatants were harvested and immunoblotted for viral capsid protein (VP1).

As expected, detection of VP1 decreased as concentrations of cycloheximide increased (Fig. 6B). We therefore investigated recovery of the assembly-deficient VP1-I194V mutant by treating transfected L cells with sublethal concentrations of cycloheximide at 1.5 h posttransfection. Cells were lysed and clarified, and infectious titers of supernatant samples were determined by plaque assays. Our data show that treatment with cycloheximide could not recover the assembly-deficient VP1-I194V mutant (Fig. 6C). Therefore, it appears that recovery of the assembly-deficient VP1-I194V mutant by 2A^pro^ cannot be replicated by partial pharmacological inhibition of protein translation.

## DISCUSSION

Virions must be sufficiently stable to protect their genome from environmental damage but flexible enough to allow the conformational changes required to deliver the genome into a new host cell (7, 39). It is likely that changes to this balance, which might occur during adaptation to unusual environmental stress, will be acquired at a cost to overall fitness (1, 3).

Previously, we reported that the thermal selection of PV-1 at increasing temperatures of 51°C, 53°C, and 57°C resulted in virus populations that consistently maintained two VP1 mutations (i.e., I194V and V87A). We further showed by site-directed mutagenesis of a wt infectious clone that a combination of VP1-I194V and VP1-V87A increased the thermal stability of the PV-1 capsid, while VP1-I194V alone abrogated virion production (25). It has been shown that VP1-I194 in all PV serotypes (or the equivalent VP1-I192 in PV-3) plays an important role in acquiring resistance to a pocket-binding antiviral compound, V-073 (40, 41). Additionally, VP1-V87A has been reported to confer heat resistance to PV-1 (42). Previously, we showed that the selected population containing both VP1 mutations (I194V and V87A) was more thermally stable than the wt infectious clone (25). Our findings suggested that VP1-I194V evolved to complement VP1-V87A, at a cost to fitness. This was, however, partially compensated for by two nonstructural protein mutations within the 2A^pro^ region of the genome (2A-I99V and 2A-G102R). We found that both sets of VP1 heat resistance and 2A^pro^ mutations were maintained together as consensus during further rounds of selection (Fig. 1B and C), suggesting their importance in maintaining the thermostable phenotype. Here, we sought to understand the functional and biological consequences of the 2A^pro^ mutations during adaptation.

Multiple roles for 2A^pro^ have been reported, which include antagonizing host immune responses (13), prolonging viral RNA translation (18, 19), stabilizing replicating RNA (22), enhancing RNA synthesis (21), and initiating morphogenesis (5). Thus, 2A^pro^ has been suggested to play a regulatory role in the PV life cycle (13). The autocatalytic *cis*-cleavage activity of 2A^pro^ occurs cotranslationally with high efficiency (Fig. 2B), while further polyprotein processing by 3C^pro^ occurs much less efficiently (26). The proteolytic activity of 2A^pro^ can be affected by mutations in the catalytic triad (14, 15) or via highly conserved cysteine and histidine residues that are known to maintain structural integrity (16, 27). Although the selected 2A^pro^ mutations did not involve any of these residues, we hypothesized that proximity of 2A-G102R to a catalytic residue (i.e., C109) could affect catalytic activity.

The cotranslational cleavage of P1/2A occurs rapidly during normal virus replication. This appeared to be slower in the rabbit reticulocyte T_N_T assays used here, which have also been reported to restrict *in vitro* translation of poliovirus RNA (43). However, T_N_T assays provide a useful platform to investigate posttranslational cleavage kinetics of PV. Using this approach to investigate the proteolytic efficiency of the selected 2A^pro^ mutations, we observed that 2A-I99V had no effect on the *cis* cleavage of the predefined P1-2A construct; however, 2A-G102R individually and in combination with 2A-I99V slowed the *cis* cleavage of P1-2A. We therefore hypothesized that the slower processing could have resulted in downstream effects on other aspects of the viral life cycle, such as delayed release of P1 and 2BC, which could influence capsid assembly and/or genome replication.

Using the HeLa cell-free system, we were able to investigate the *cis* cleavage of the entire polyprotein by the mutant 2A^pro^. This showed an accumulation of large precursors, P1-P2 and P1-2A, with an abundance of the former. Both of these precursor proteins were slowly processed by the protease 3C/3CD^pro^. The replication of PV has been shown to involve the formation of a membrane-bound complex of 2B and 2C (9, 10) and 3A (11). We speculate that the delayed release of 2BC may have resulted in delayed recruitment of 2B and 2C to this replication complex (9, 10), and thus, genome replication was significantly affected (Fig. 4). Given the global change to the order in which the precursor protein is cleaved, it is perhaps surprising that virus replication is still supported. Such global changes to how polyproteins are processed could provide a mechanism by which positive-sense RNA viruses can quickly adapt to selection pressures via changing the repertoire and order of proteins produced through protease mutations.

Although we do not fully understand the role of 2A-I99V, the maintenance of this mutation together with G102R within the consensus sequence prompted our investigation of the combined effects of both mutations on the VP1-I194V capsid mutant. Our results suggested that the 2A^pro^ mutations compensated for the assembly-deficient VP1-I194V mutant (25) (Fig. 5). Together, our data suggest that a combination of 2A-I99V and 2A-G102R modulated the *cis*-mediated cleavage of P1 (9, 10) and significantly decreased the rates of genome replication to alter the dynamics of virion assembly and overcome the otherwise deleterious effect of VP1-I194V capsid mutation (44). However, further investigation will be required to fully understand the mechanisms through which the 2A^pro^ mutations affected replication of the genome.

Assembly of the PV capsid is a complex process requiring highly efficient translation, which can be lethally affected by deoptimization of the capsid codons (45). In our previous study we reported that thermal selection of PV-1 (generating variants with thermally stable capsids) occurred at a cost to virion assembly which was due to a VP1-I194V mutation. We further speculated that a capsid-stabilizing mutation, VP1-V87A, could ameliorate the assembly deficiency effects of VP1-I194V through unknown mechanisms (25). Here, we have traced the selection of VP1-I194V to the coexistence of a pair of second-site (compensatory) mutations within 2A^pro^, which may have sustained VP1-I194V within the quasispecies. The role of V87A is unclear, but the coexistence of I194V and V87A in all subsequently selected heat-resistant viruses suggests a functional link between these two mutations. Our findings also showed that partial inhibition of translation of the polyprotein using cycloheximide could not recover the assembly-deficient VP1-I194V mutant (Fig. 6); however, reduction of the rate of polyprotein processing by the mutations (2A-I99V and 2A-G102R) appeared to favor assembly. Together our findings suggest that recovery of the assembly-deficient VP1-I194V mutant by 2A^pro^ may have involved a chaperone-like activity provided by 2A^pro^.

Chaperones prevent aggregation of proteins in highly crowded cellular environments (30, 31) and facilitate viral capsid assembly. Host-encoded chaperones have been reported for enteroviruses (including PV [46]), hepatitis B virus (47), and bacteriophages (48, 49). Furthermore, while some virally encoded proteins have also been reported to have chaperone-like activities that facilitate capsid assembly, e.g., the capsid-associated protein 80 (p80) of African swine fever virus (50), the T antigen (TAg) of simian virus 40 (SV40) (51), and the nonstructural protein 40 (NSP40) of herpes simplex virus (52). Early-stage morphogenesis of PV is facilitated by the host-encoded chaperone Hsp70 (46), while virally encoded 2C facilitates later stages of morphogenesis of virions (i.e., genome encapsidation) (12, 53–55).

Owing to their small genome sizes, virus-encoded proteins such as 2A^pro^ have been shown to perform multiple roles. Our study provides the first suggestion that PV 2A^pro^ could act as a chaperone-like protein and suggests regulatory roles for 2A^pro^ in maintaining the balance between virus fitness and virion stability that allows the emergence of heat-resistant PV-1 variants.

## MATERIALS AND METHODS

### Antibodies.

Rabbit polyclonal anti-glyceraldehyde-3-phosphate dehydrogenase (anti-GAPDH) (G9545) and anti-rabbit polyclonal (A0545) antibodies were commercially sourced from Sigma-Aldrich (now Merck), Germany. Mouse anti-poliovirus 1 (anti-VP1) monoclonal antibody (MAb) 8560 was commercially sourced from Millipore (now Merck), Germany.

### Cell lines and virus propagation.

HeLa and mouse L cells were obtained from the National Institute of Biological Standards and Control, UK. Viruses were propagated by standard methods. Infectivity titers were determined by plaque assays using HeLa monolayer cells (57) and expressed as PFU per milliliter.

### Viral genome extraction and sequencing.

RNA was extracted from virion samples using guanidinium thiocyanate-phenol-chloroform (58). The sequence from the 5′ UTR to the end of the structural (P1) coding region (i.e., nucleotide positions 1 to 3385 within the genome) was reverse transcribed and PCR amplified using previously described downstream (P1Rev) and upstream (P1Fwd) primers (5′-CTTGGCCACTCAGGATGATT-3′ and 5′-TTAAAACAGCTCTGGGGTTGTAC-3′, respectively) (25). Both structural and nonstructural regions (i.e., nucleotide positions 1 to 7407 within the genome) were reverse transcribed and PCR amplified using downstream (P3Rvs) and upstream (P1Fwd) primers 5′-GTATGACCCAATCCAATTCGACT-3′ and 5′-TTAAAACAGCTCTGGGGTTGTAC-3′, respectively. PCR amplicons were sequenced by Sanger methods (59) and cloned into the pGEM-T Easy vector, and individual colonies were sequenced (60). Primer sequences are available on request.

### Recombinant DNA techniques.

Poliovirus was recovered from an infectious clone sourced from Bert Semler, University of California. The full PV-1 (Mahoney) genome was cloned into vector pT7Rbz, which incorporates a T7 RNA polymerase promoter to allow *in vitro* RNA synthesis and a ribozyme overhang (61). For the *in vitro* assay, the PV-1 P1/2A precursor was cloned into vector pcDNA 3.1(+) with a Kozak sequence. Several subgenomic constructs were designed and incorporated into a pcDNA 3.1(+) vector. These included pcDNA-P1-2A (which has a wt P1-2A), pcDNA-P1-2A_I99V_ (which has a wt P1 and an I99V mutation introduced into 2A), pcDNA-P1-2A_G102R_ (which has a wt P1 and a G102R mutation introduced into 2A), pcDNA-P1-2A_I99V/G102R_ (which has a wt P1 and a combination of I99V and G102R mutations introduced into 2A), pcDNA-P1-P2 (i.e., a noncleavable P1-P2 construct with a 2A-C109A mutation introduced), and pcDNA-P1-2A (i.e., a noncleavable P1-P2 construct with a 2A-C109A mutation introduced). All mutations were introduced by SDM (62).

### Real-time subgenomic replicon replication assay.

Using a previously described PV-1 (strain Mahoney) cDNA clone (pT7RbzPV-1) (25), a subgenomic replicon was designed and termed pRepPV1-wt. Here, the P1 capsid precursor was replaced with the green fluorescent protein (GFP)-coding sequence from Ptilosarcus gurneyi. The modified pT7RbzGFP replicon incorporated the PV-1 5′ UTR, residues 1 to 23 of VP0, ptGFP, the last 25 residues of VP1, the PV P2 region, the P3 region, and the 3′ UTR, followed by the rest of pT7Rbz. Both 2A^pro^ mutations were introduced into pRepPV1 individually (to create pRepPV1-2A_I99V_ and pRepPV1-2A_G102R_) or in combination (to create pRepPV1-2A_I99V/G102R_) by SDM (62). pRepPV1 was linearized using EcoRI and RNA transcribed *in vitro* by T7 RNA polymerase (63). One-microgram aliquots of RNA transcripts were transfected into HeLa cells using Lipofectin. Replicon replication was assessed in real time as GFP expression by live-cell imaging within the IncuCyte dual-color Zoom, which is an automated phase-contrast and fluorescence microscope within a 37°C humidifying CO_2_ incubator. Cells were monitored every 30 min posttransfection for up to 24 h. Nine images per well were taken at each time to measure the GFP object counts per well, as well as the total fluorescence intensity per well using an integrated software, and analyzed according to standard methods (64).

### *In vitro* T_N_T assay.

**(i) Rabbit reticulocyte lysates.** Subgenomic PV-1 constructs were cloned into the pcDNA 3.1(+) vector individually (i.e., pcDNA-P1-2A, pcDNA-P1-2A_I99V_, and pcDNA-P1-2A_G102R_) or in combination (i.e., pcDNA-P1-2A_I99V/G102R_) and expressed in the T_N_T Quick coupled transcription/translation (T_N_T) system (Promega) in the presence of [^35^S]Cys-Met according to the manufacturer’s protocol. Following an incubation period of 90 min at 30°C, further incorporation of ^35^S was prevented by addition of excess unlabeled Cys-Met, and samples were taken at intervals of 30 min. Proteins were separated by SDS-PAGE (65) and detected by autoradiography (66) and phosphorimaging (67).

**(ii) HeLa cell-free extracts.** HeLa cell (S10) extracts and initiation factor (IF) fractions were gifted by David Barton, University of Colorado and also were prepared according to standard protocols (33, 68). Reaction mixtures contained 50% (vol/vol) S10, 20% (vol/vol) IF, 10% (vol/vol) 10× reaction buffer (10 mM ATP, 2.5 mM GTP, 2.5 mM CTP, 2.5 mM UTP, 600 mM KCH_3_CO_2_, 300 mM creatine phosphate, 4 mg/ml creatine kinase, and 155 mM HEPES-KOH [pH 7.4]), 3.2μg T7 RNA transcripts, and 38 μCi [^35^S]Cys-Met. Reaction mixtures were incubated at 34˚C for 2 h and chased with excess amounts of unlabeled Cys-Met. Sample proteins were separated by 8% SDS-PAGE, and [^35^S]Cys-Met-labeled proteins were detected by standard protocols for autoradiography (66) and phosphorimaging (67).

### Virus recovery from infectious clones.

A total of 2.5 × 10^6^ mouse L cells or HeLa cells were transfected with 5 μg of RNA transcripts using Lipofectin according to the manufacturer’s protocols. Transfected cells were incubated at 37°C for 16 h and cell harvests titrated for infectivity after disruption by freeze-thawing.

### Cycloheximide cytotoxicity assay.

The compound 4-(2-hydroxyethyl) piperidine-2,6-dione, also known as cycloheximide, was commercially sourced from Sigma-Aldrich (now Merck), Germany.

Cell culture 96-well vessels were seeded to 4 × 10^4^ cells per well. Triplicate wells of seeded cells were treated with cycloheximide at increasing concentrations. Cells were incubated at 37°C under 5% CO_2_ for 24 h and assayed for toxicity using a 3-(4,5-dimethylthiazol-2-yl)-5-(3-carboxymethoxyphenyl)-2-(4-sulfophenyl)-2H-tetrazolium (MTS) assay kit (Promega CellTiter 96 Aqueous One Solution Cell Proliferation) according to the manufacturers’ protocol.

### Translation inhibition assays.

Mouse L cells were transfected with 3 μg T7 RNA transcripts of a PV-1 infectious clone, pT7Rbz. At 1.5 h posttransfection, cycloheximide was added to the indicated concentrations. Cells were incubated at 37°C under 5% CO_2_ and harvested after 24 h. Supernatants from each well were clarified by centrifugation at 4,000 rpm for 2 min. Cells were trypsinized, washed, and lysed using radioimmunoprecipitation assay (RIPA) buffer (50 mM Tris-Cl [pH 7.4], 150 mM NaCl, 1% NP-40, 0.5% sodium deoxycholate, 0.1% SDS). Proteins were separated by 8% SDS-PAGE (65) and immunoblotted using an anti-VP1 MAb (69) by standard protocols.

### Densitometry.

Scanned images were analyzed by ImageJ (70) version 1.47t according to standard procedures. Briefly, scanned image blots or phosphoscreened autoradiographs were saved in the Tagged Image File Format (TIFF). Selected bands of interest were individually selected, and pixilated band intensities were quantified according to software algorithms.

### Genome sequences and alignment.

Reference genome sequences of the following viruses were sourced from GenBank and downloaded in the FASTA format: coxsackievirus A1 (CVA-1) (AGI61097.1), echovirus 1 (EV1) (AAC63944.2), PV-1 (P03300), PV-2 (AAA46912.1), PV-3 (AAN85444.1), CVA-2 (ANQ47259.1), enterovirus 94 (EV-94) (ABL61316.1), bovine enterovirus 1 (BEV-1) (P12915.3), BEV-2 (ADU34211.1), EV-G1 (AIA21703.1), SV4 (AAL69631.2), human rhinovirus A (HRV-A) (CAA26181.1), HRV-B (ACK37380.1), and HRV-C (ABK29455.2). Alignment of sequences was carried out at the protein level using the MUltiple Sequence Comparison by Log-Expectation (MUSCLE) algorithm of the CLC Sequence Viewer version 7.8.1 software.

### Statistical analysis.

Statistical analysis of mutants against the wt was done with Student *t* tests using GraphPad Prism version 7.01 for Windows (GraphPad Software, La Jolla CA).
